# Supplementary material for: Role of transoesophageal echocardiography in detecting patent foramen ovale in stroke patients aged ≤60 years: A retrospective study
Source: PLoS One. 2020 Nov 30;15(11):e0242885. doi: 10.1371/journal.pone.0242885 (PMC7703908; doi:10.1371/journal.pone.0242885)
Supplement: Attachment — Submitted filename: Response to Reviewers.docx [file pone.0242885.s002.docx]

September 30, 2020

Editorial Board

*PLOS One*

To the Editors,

I wish to re-submit the manuscript titled, ‘Role of transoesophageal echocardiography in detecting patent foramen ovale in stroke patients aged <60 years: A retrospective study’. The manuscript ID is PONNEE--DD--2200—221133440.

I would like to thank you and the reviewers for your thoughtful suggestions and insights. The manuscript has benefited from these insightful suggestions. I look forward to working with you and the reviewers to move this manuscript closer to publication in *PLOS One.*

The manuscript has been rechecked and the necessary changes have been made in accordance with the reviewers’ suggestions. The responses to all comments have been prepared given below. Additionally, a data availability statement as been prepared on a separate document.

Thank you for your consideration. I look forward to hearing from you.

Sincerely,

Reabal Najjar

Australian National University Medical School

Canberra, ACT 2605, Australia

Tel.: +61 450 722 549

Email: reabal.najjar@anu.edu.au

**Responses to the Reviewer’s Comments**

**Title**

**Comment 1:** Although etiologies of ischemic strokes occurring in people aged <60 years have important differences when compared with older people, the title “young stroke patient” can be considered misleading, since the “traditional” cut-off for deﬁning a young person is usually somewhere between 40-50 years. I would advise to be more descriptive by saying something like “… foramen ovale in stroke patients aged <60 years: A retrospective…”. This may be important, since the proportion of patients aged 51-59 was 55% in this data set. It is true that some other authors have previously used the term “young” for people aged >60 years (even in RCTs), but it has been arguable in the “strokeologists” community, given the current WHO deﬁnitions.

**Response:** The title has been changed in accordance with your comment (page 1, lines 1-3).

**Abstract**

**Comment 2:** It would be interesting to declare the other TTE and TOE ﬁndings, to make possible calculating the proportion of PFOs among the other putative cardioembolic stroke etiologies. This may be important, since PFO in this age group may be an important putative etiology.

**Response:** ASD findings have been provided in the Results section of the Abstract (page 2, line 27).

**Comment 3**: The sentence “The rate of PFO detection by TOE was correlated with younger age” needs statistical support, since correlation (not confounding with “association”) is a speciﬁc type of statistical analysis (i.e., either Pearson’s or Spearman’s).

**Response:** In this sentence, ‘correlation’ has been changed to ‘association’ (page 2, lines 32).

**Comment 4:** Since PFO is mainly (although not solely) associated with AIS through paradoxical embolism, it is pertinent t ask, How many venous thrombotic events were identiﬁed among the whole cohort and among “cryptogenic” of “Known cause” patients? This can be deduce from the manuscript body, but it may be advisable to include this important information in the Abstract.

**Response:** This important information has been included in the Abstract (page 2, lines 26-28).

**Comment 5:** In the Abstract’s conclusion is better to say “In stroke patients aged 18–60 years…”.

**Response:** Your suggested change has been made in the Abstract (page 2, line 32).

**Comment 6:** The statement “the effectiveness of PFO closure has been established 29 over medical treatment in reducing future reoccurrence of ischaemic stroke” lacks of supports in Results/Findings, i.e., it is not possible to conclude in such a sentence with the support of ﬁndings provided in the Abstract.

**Response:** The conclusion of the Abstract has been changed to better reflect the study’s results/findings (page 2, lines 32-35).

**Comment 7:** The authors provide results such as NNT in conclusions, but not in Results. This is a little odd, since conclusions is the part of the Abstract where authors refer to ﬁndings provided ins Results section.

**Response:** The NNT results have been moved from the Conclusions to the Results (page 2, lines 30-31).

**Comment 8:** I agree with the authors in that TOE may be better than TTE to detect PFO, but no ﬁndings where presented in the Abstract that forcefully support this notion.

**Response:** Further findings have been added in the Abstract in order to support TOE > TTE (page 2, lines 28-30).

**Comment 9:** In brief, more results in Abstract may make this paper more citable.

**Response:** More results have been added to the Abstract to make this paper more citable (page 2, lines 25-31).

**Methods**

**Comment 10:** It is necessary to declare whether in this medical center some stroke patients are admitted outside the stroke unit, and if so, how this characteristic may bias the clinical practice in searching for AIS etiology.

**Response:** This change has been added to reflect that all patients in the centre presenting with a stroke were admitted to the stroke unit (page 4, line 128).

**Comment 11:** It is not completely clear why some patients received either TTE or TOE. Did some patients received ﬁrstly TTE and later TOE to actively ﬁnd a PFO? What were the factors used in clinical practice to decide between TTE or TOE in this center?

**Response:** This information has been added to elaborate on the choice of TTE or TOE (pages 5-6, lines 179-198).

**Comment 12:** The etiology categories are not traditional. I would rather recommend to use TOAST or ASCOD classiﬁcation to comply with “traditional” language or understanding. After this, the “new” classiﬁcation proposed by the authors can also be used to make the authors’ point.

**Response:** The traditional TOAST classification has been used along with our simplified version to address our point (page 5, lines 169-173).

**Results**

**Comment 13:** I would rather change “TTE and TOE rates” to “TTE and TOE usage rates”.

**Response:** Your suggested change has been made (pages 8-9, lines 262, 272, 274-276, 280).

**Comment 14:** The ﬁgure 2 can be eliminated, since only represents proportions that can be included only as text.

**Response:** The pie chart used for stroke aetiology proportions has been deleted.

**Comment 15:** The ﬁgure 3 has a legend that seems confusing. ROCs plot sensitivity Vs 1-speciﬁcity of a diagnostic tool. This ROC graphic has been used by the authors plotting age as it were a diagnostic tool, but in the Results authors state that this plot is about the performance of TTE and TOE, but this is not the case. TTE or TOE provides either a “negative” (i.e., 0) or positive (i.e.,1) test result that would produce a different ROC pattern. I would not use age as a predicting/diagnosting factor to detect PFO.

**Response:** Thank you for pointing this out. The ROC curve has been removed.

**Comment 16:** The authors are not providing actual diagnostic performance data (i.e., sens, spec, PPV, NPV, LR+, LR-, accuracy, Youden’s index, etc.) to make the point that TOE performed better than TTE in detecting PFOs. This simply was not demonstrated in this study, so that claim cannot be supported.

**Response:** The diagnostic performance data, sensitivity, specificity, predictive values, and likelihood ratios, have been provided in a new table (Table 3) for comparison between TTE and TOE.

**Discussion**

**Comment 17:** A limitations section is needed for better interpretation of this hypothesis-generating study, especially with respect to the inherent bias of a retrospective study, and an analysis or explanation about what determined too practice either TTE or TOE in this set of patients.

**Response:** The limitations section has been expanded and elaborated on to further reflect the weaknesses of this retrospective study (page 14, lines 503-514).

**Comment 18:** To better answer the scientiﬁc question about which is better, TTE or TOE, the best way to do it is to use TTE AND TOE in ALL patients. If some patients received either TTE or TOE, the performance of either imaging technique may be inﬂuenced by patients’ factors. Please discussed more on that.

**Response:** We have discussed this scientific question and mentioned the use of TTE and TOE in all patients (page 13, lines 483-487).

**Comment 19:** Please comment more about the very low frequency of deep vein thrombosis as compared with PFO, and the limitation of assigning causality by only ﬁnd a PFO without active or strong identiﬁcation of paradoxical embolism.

**Response:** We have addressed DVT vs. PFO and the limitation in assigning causality (page 14, lines 507-510).
