# Supplementary material for: Role of transoesophageal echocardiography in detecting patent foramen ovale in stroke patients aged ≤60 years: A retrospective study
Source: PLoS One. 2020 Nov 30;15(11):e0242885. doi: 10.1371/journal.pone.0242885 (PMC7703908; doi:10.1371/journal.pone.0242885)
Supplement: Attachment — Submitted filename: Response to Reviewers.docx [file pone.0242885.s003.docx]

October 31, 2020

Editorial Board

*PLOS One*

To the Editors,

I wish to re-submit the manuscript titled, ‘Role of transoesophageal echocardiography in detecting patent foramen ovale in stroke patients aged <60 years: A retrospective study’. The manuscript ID is PONNEE--DD--2200—221133440.

I would like to thank you and the reviewers for your thoughtful suggestions and insights. The second round of revisions has been completed. I look forward to working with you and the reviewers to move this manuscript closer to publication in *PLOS One.*

The manuscript has been rechecked and the necessary changes have been made in accordance with the reviewers’ suggestions. The responses to all comments have been prepared given below.

Thank you for your consideration. I look forward to hearing from you.

Sincerely,

Reabal Najjar

Australian National University Medical School

Canberra, ACT 2605, Australia

Tel.: +61 450 722 549

Email: reabal.najjar@anu.edu.au

**Responses to the Reviewer’s Comments**

**ABSTRACT**

COMMENT 1: -In the new Abstract the authors state that “The number needed to treat (NNT) to prevent the occurrence of an ischaemic stroke through PFO closure was 30.” However, since this is an observational analysis, real NNTs can be only inferred from RCTs, but not direct calculation can be obtained since no actual PFO closure was performed and compared with no closure to obtain real NNTs. Nonetheless, this calculation can be useful to the readers as a hypothetical estimation. Maybe the authors can lower the tone of this claim by saying that NNT to prevent a putative AIS recurrence through PFO closure was estimated at 30.

**Response:** The claim has been adjusted to reflect the hypothetical estimation (page 2, line 31).

COMMENT 2: -There is no need to declare the NNT acronym in Abstract if it will not be used again in the text.

**Response:** The acronym has been removed (page 2, line 30).

COMMENT 3: -In the previous Comment 4 (about the suggestion of including how many systemic venous thromboembolic events were identified in these patients) the authors responded “This important information has been included in the Abstract (page 2, lines 26-28).”, however, no information about the number of venous thrombotic events can be read from this new version of the Abstract. This may be of interest to the readers, because PFO is putatively associated with AIS through paradoxical embolism arising from thrombosis in the venous side of the circulation.

**Response:** Apologies, the number of venous thromboembolic events have been added (page 2, lines 26-27).

**RESULTS**

COMMENT 4: -I would recommend that the new Table 3 include the 95% CI for the estimations. This can be rapidly and freely obtained by using the following web-based tool:
<https://ebm-tools.knowledgetranslation.net/calculator/diagnostic/>

**Response:** The 95% CI for the estimations were added in table 3 (page 10, lines 214-218).
