# Supplementary material for: Role of transoesophageal echocardiography in detecting patent foramen ovale in stroke patients aged ≤60 years: A retrospective study
Source: PLoS One. 2020 Nov 30;15(11):e0242885. doi: 10.1371/journal.pone.0242885 (PMC7703908; doi:10.1371/journal.pone.0242885)
Supplement: Attachment — Submitted filename: Proof and Print Reviewer Comments.pdf [file pone.0242885.s001.pdf]

Reviewer Recommendation and Comments for Manuscript Number PONE-D-20-21340

Role of transoesophageal echocardiography in detecting patent foramen ovale in young stroke patients: A retrospective study.

Original Submission
Erwin Chiquete, M.D., Ph.D.

Back | Edit Review | Print | Submit Review to Editorial Office

Recommendation: Major Revision

**Custom Review Question(s):**	**Response**
**Comments to the Author**<br><br>1. Is the manuscript technically sound, and do the data support the conclusions?<br><br>The manuscript must describe a technically sound piece of scientific research with data that supports the conclusions. Experiments must have been conducted rigorously, with appropriate controls, replication, and sample sizes. The conclusions must be drawn appropriately based on the data presented.	Partly
2. Has the statistical analysis been performed appropriately and rigorously?	No
3. Have the authors made all data underlying the findings in their manuscript fully available?<br><br>The PLOS Data policy requires authors to make all data underlying the findings described in their manuscript fully available without restriction, with rare exception (please refer to the Data Availability Statement in the manuscript PDF file). The data should be provided as part of the manuscript or its supporting information, or deposited to a public repository. For example, in addition to summary statistics, the data points behind means, medians and variance measures should be available. If there are restrictions on publicly sharing data—e.g. participant privacy or use of data from a third party—those must be specified.	No
4. Is the manuscript presented in an intelligible fashion and written in standard English?<br><br>PLOS ONE does not copyedit accepted manuscripts, so the language in submitted articles must be clear, correct, and unambiguous. Any typographical or grammatical errors should be corrected at revision, so please note any specific errors here.	Yes
5. Review Comments to the Author<br><br>Please use the space provided to explain your answers to the questions above. You may also include additional comments for the author, including concerns about dual publication, research ethics, or publication ethics. (Please upload your review as an attachment if it exceeds 20,000 characters)	I have the opportunity to review the Najjar, et al. paper titled “Role of transoesophageal echocardiography in detecting patent foramen ovale in young stroke patients: A retrospective study”. I would like to thank this honor.<br><br>This paper has valuable data, although with the inherent limitations of a retrospective analysis, especially about the bias that could indicate TOE in a given patient. Nonetheless, I have some comments.<br><br>COMMENTS<br><br>Title<br>-Although etiologies of ischemic strokes occurring in people aged <60 years have important differences when compared with older people, the title “young stroke patient” can be considered misleading, since the “traditional” cut-off for defining a young person is usually somewhere between 40-50 years. I would advise to be more descriptive by saying something like “… foramen ovale in stroke patients aged <60 years: A retrospective…”. This may be important, since the proportion of patients aged 51-59 was 55% in this data set. It is true that some other authors have previously used the term “young” for people aged >60 years (even in RCTs), but it has been arguable

in the “strokeologists” community, given the current WHO definitions.

Abstract
-It would be interesting to declare the other TTE and TOE findings, to make possible calculating the proportion of PFOs among the other putative cardioembolic stroke etiologies. This may be important, since PFO in this age group may be an important putative etiology.
-The sentence “The rate of PFO detection by TOE was correlated with younger age” needs statistical support, since correlation (not confounding with “association”) is a specific type of statistical analysis (i.e., either Pearson’s or Spearman’s).
-Since PFO is mainly (although not solely) associated with AIS through paradoxical embolism, it is pertinent t ask, How many venous thrombotic events were identified among the whole cohort and among “cryptogenic” of “Known cause” patients? This can be deduce from the manuscript body, but it may be advisable to include this important information in the Abstract.
-In the Abstract’s conclusion is better to say “In stroke patients aged 18–60 years…”.
The statement “the effectiveness of PFO closure has been established 29 over medical treatment in reducing future reoccurrence of ischaemic stroke” lacks of supports in Results/Findings, i.e., it is not possible to conclude in such a sentence with the support of findings provided in the Abstract.
-The authors provide results such as NNT in conclusions, but not in Results. This is a little odd, since conclusions is the part of the Abstract where authors refer to findings provided ins Results section.
-I agree with the authors in that TOE may be better than TTE to detect PFO, but no findings where presented in the Abstract that forcefully support this notion.
-In brief, more results in Abstract may make this paper more citable.

Methods
-It is necessary to declare whether in this medical center some stroke patients are admitted outside the stroke unit, and if so, how this characteristic may bias the clinical practice in searching for AIS etiology.
-It is not completely clear why some patients received either TTE or TOE. Did some patients received firstly TTE and later TOE to actively find a PFO? What were the factors used in clinical practice to decide between TTE or TOE in this center?
-The etiology categories are not traditional. I would rather recommend to use TOAST or ASCOD classification to comply with “traditional” language or understanding. After this, the “new” classification proposed by the authors can also be used to make the authors’ point.

Results
-I would rather change “TTE and TOE rates” to “TTE and TOE usage rates”.
-The figure 2 can be eliminated, since only represents proportions that can be included only as text.
-The figure 3 has a legend that seems confusing. ROCs plot sensitivity Vs 1-specificity of a diagnostic tool. This ROC graphic has been used by the authors plotting age as it were a diagnostic tool, but in the Results authors state that this plot is about the performance of TTE and TOE, but this is not the case. TTE or TOE provides either a “negative” (i.e., 0) or positive (i.e., 1) test result that would produce a different ROC pattern. I would not use age as a predicting/diagnosting factor to detect PFO.
-The authors are not providing actual diagnostic performance data (i.e., sens, spec, PPV, NPV, LR+, LR-, accuracy, Youden’s index, etc.) to make the point that TOE performed better than TTE in detecting PFOs. This simply was not demonstrated in this study, so that claim cannot be supported.

Discussion
-A limitations section is needed for better interpretation of this hypothesis-generating study, especially with respect to the inherent bias of a retrospective study, and an analysis or explanation about what determined too practice either TTE or TOE in this set of patients.
-To better answer the scientific question about which is better, TTE or TOE, the best way to do it is to use TTE AND TOE in ALL patients. If some patients received either TTE or TOE, the performance of either imaging technique may be influenced by patients' factors. Please discussed more on that.
-Please comment more about the very low frequency of deep vein thrombosis as compared with PFO, and the limitation of assigning causality by only find a PFO without active or strong identification of paradoxical embolism.

6. PLOS authors have the option to publish the peer review history of their article (what does this mean?). If published, this will include your full peer review and any attached files.

If you choose "no", your identity will remain anonymous but your review may still be made public.

Do you want your identity to be public for this peer review? For information about this choice, including consent withdrawal, please see our Privacy Policy.

No

Confidential to Editor

This paper has interesting data, but can improve substantially before being published.

1. Do you have any potential or perceived competing interests that may influence your review? Please review our Competing Interests policy and declare any potential interests that you feel the Editor should be aware of when considering your review. If you have no competing interests, please write "I have no competing interests."

2. Did you receive any assistance in preparing this review (e.g. from a post-doc or graduate student)? If yes, please include their name below.

3. If accepted, do you think this submission should be highlighted on the PLOS ONE website? PLOS ONE does not evaluate manuscripts based on perceived significance or readership. We aim to provide tools for readers to filter and evaluate our publications. (optional)

No

Back | Edit Review | Print | Submit Review to Editorial Office
